# Metastatic malignant phyllodes tumor of the breast with rapid progression and subsequent response to immunotherapy: a case report

**DOI:** 10.3389/fonc.2026.1778276

**Published:** 2026-05-26

**Authors:** Lei Cao, Junxia Hu, Xueyuan Mao, Yinggang Xu, Wenjing Ma, Xinyuan She, Ye Zhang, Peng Yang, Xiangxin Zheng

**Affiliations:** 1 Department of Oncology, Jiangsu Province (Suqian) Hospital, Suqian, Jiangsu, China; 2 Department of Oncology, Suqian Clinical Medical College of Jiangsu University, Suqian, Jiangsu, China; 3 Department of Pathology, Jiangsu Province (Suqian) Hospital, Suqian, Jiangsu, China; 4 Department of Breast and Thyroid Surgery, Jiangsu Province (Suqian) Hospital, Suqian, Jiangsu, China

**Keywords:** immune checkpoint inhibitor, maintenance therapy, malignant phyllodes tumor, metastatic, multitarget tyrosine kinase inhibitor, PD-L1

## Abstract

**Background:**

Malignant phyllodes tumors (MPTs) of the breast are rare fibroepithelial neoplasms characterized by aggressive clinical behavior and limited evidence guiding systemic therapy in the metastatic setting. Immune checkpoint inhibitors (ICIs) have demonstrated activity in several sarcoma subtypes; however, their role in MPTs of the breast remains poorly defined.

**Case presentation:**

A 53-year-old female patient underwent modified radical mastectomy for a rapidly enlarging left breast mass. Pathology revealed a 16.0 × 15.0 × 9.5 cm malignant phyllodes tumor with lymphovascular invasion (pT4N0). Despite the presence of an 8-mm pulmonary nodule at baseline, multiple bilateral lung metastases developed shortly after surgery. Following eight cycles of ifosfamide plus epirubicin chemotherapy, tumor burden was reduced by more than 90%. The patient subsequently received anlotinib as maintenance therapy; four months later, new hepatic and colorectal metastases emerged while the pulmonary lesions remained stable. Second-line sintilimab-based combination therapy achieved a marked partial response, with significant regression of the abdominal lesions, liquefactive necrosis of the hepatic metastases, and complete relief of abdominal pain.

**Conclusion:**

This case illustrates the diagnostic and therapeutic complexity of metastatic malignant phyllodes tumors. Although first-line chemotherapy produced marked pulmonary tumor regression, subsequent anlotinib maintenance therapy did not prevent disease progression. In this PD-L1–positive tumor (CPS 10), sintilimab-based combination therapy yielded a marked clinical and radiographic response, supporting further investigation of ICI-based therapy in selected patients with metastatic MPT.

## Introduction

Malignant phyllodes tumors (MPTs) are rare fibroepithelial neoplasms of the breast, accounting for less than 1% of all breast tumors (1, 2). MPTs typically present as rapidly enlarging, large breast masses, whereas lymph-node metastasis is uncommon (usually <5%) (3, 4). When distant metastases occur, they show a predilection for specific sites—most commonly the lungs, followed by bone and liver, with pulmonary metastases occurring most frequently (5).

Due to the rarity of MPTs, large randomized trials are scarce, and no standard systemic therapy exists for metastatic disease. Treatment strategies are often extrapolated from soft tissue sarcoma (6–8). Anthracycline combined with ifosfamide chemotherapy is a first-line regimen for soft-tissue sarcoma, and several studies in soft-tissue sarcoma have supported the use of antiangiogenic agents, such as anlotinib, as switch-maintenance therapy following first-line chemotherapy (9–11). In parallel with these conventional therapeutic approaches, immune checkpoint inhibitors have emerged as a promising therapeutic option across various sarcoma subtypes (12, 13). However, despite this progress, robust clinical data supporting the efficacy of ICIs in MPTs remain extremely limited, and the predictive molecular biomarkers that could guide individualized ICI treatment for this unique tumor have not yet been definitively identified.

We report the clinical course of a 53-year-old woman with MPT of the left breast who developed bilateral pulmonary metastases early after surgery and achieved a partial response to first-line chemotherapy. To limit cumulative anthracycline toxicity while maintaining disease control, she was transitioned to maintenance oral anlotinib, a multitarget tyrosine kinase inhibitor with established activity in soft-tissue sarcoma. Following 4 months of anlotinib maintenance treatment, new hepatic and colonic metastatic lesions emerged while pulmonary disease remained stable, leading to a designation of progressive disease (PD) by RECIST criteria. A sintilimab-based combination therapy was subsequently initiated, achieving a significant partial response. We analyze disease progression and therapeutic response, with a particular focus on the efficacy of ICIs-based second-line therapy, in the context of the complete clinical data and the available literature, with the aim of providing clinically relevant insights to inform the management of metastatic MPTs.

## Case description

**Figure 1:**
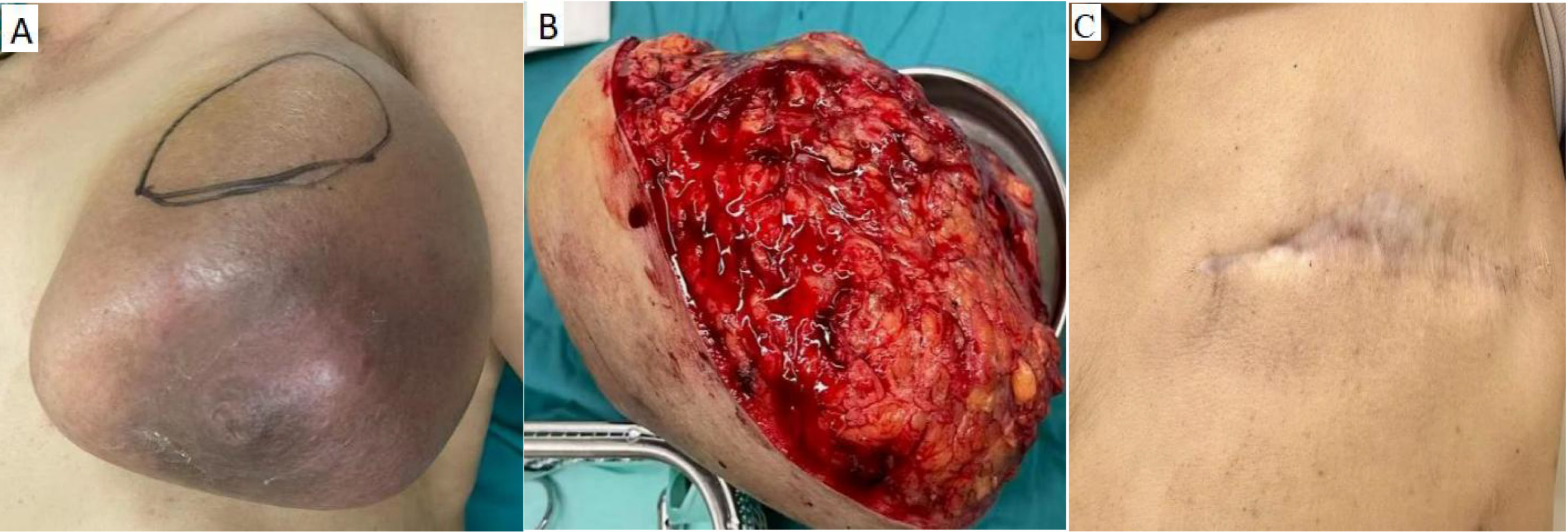
**(A)** Preoperative mass; **(B)** tumor mass resection; **(C)** current status of the chest wall.

In February 2025, a 53-year-old woman (ECOG performance status 0) presented with a rapidly enlarging giant left breast mass (**Figure 1**). She reported progressive breast enlargement and local pain, without skin ulceration, nipple discharge, fever, weight loss, or respiratory/abdominal symptoms. The patient expressed distress from rapid tumor growth and strongly requested immediate surgery to relieve symptoms, considering the prolonged waiting time for pathologic results from needle biopsy. Additionally, given the extremely thin overlying skin and the risk of persistent ulceration with poor healing associated with biopsy, preoperative core needle biopsy and immunohistochemical testing were not performed. Preoperative computed tomography revealed a small nodule in the right lung measuring 8 × 6mm. Biopsy of this pulmonary lesion was not performed due to its small size and deep location, the high risk of pneumothorax associated with non-vertical puncture, and the patient’s strong refusal of invasive procedures.

**Figure 2:**
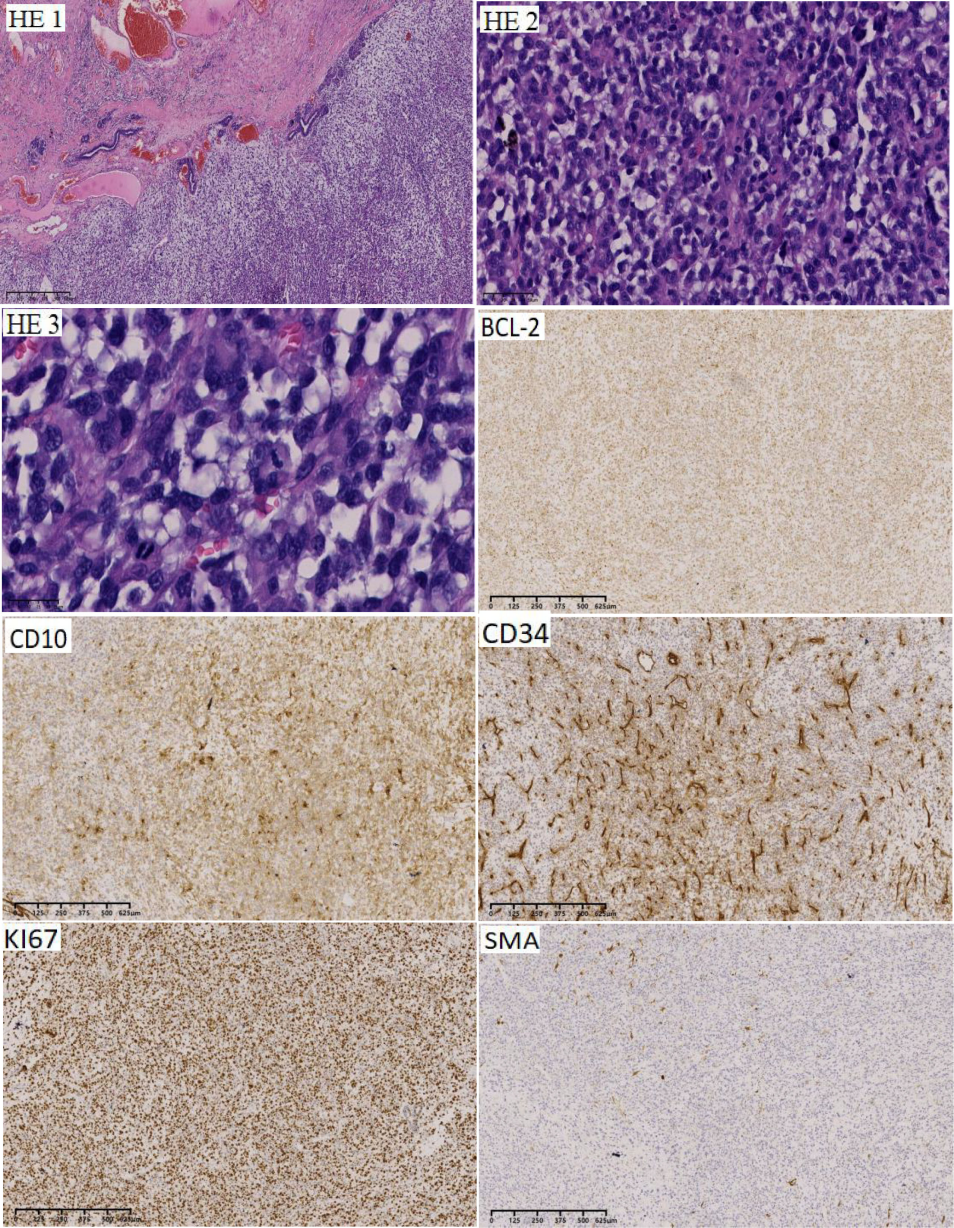
Histopathological and immunohistochemical features of the malignant phyllodes tumor. HE 1, Low-power view (HE staining, ×40) showing the infiltrative tumor border and phyllodes architecture. HE 2, High-power view (HE staining, ×400) showing marked stromal cellularity and atypia. HE 3, High-power view (HE staining, ×800) demonstrating nuclear atypia and a mitotic figure. Immunohistochemical staining (×40): Ki67(+,80%),SMA(-),CD34(+),Bcl-2(+),and CD10(+). Abbreviations: HE, hematoxylin and eosin; Ki-67, proliferation marker; SMA, smooth muscle actin; CD34, cluster of differentiation 34; Bcl-2, B-cell lymphoma 2; CD10, cluster of differentiation 10.

**Figure 3:**
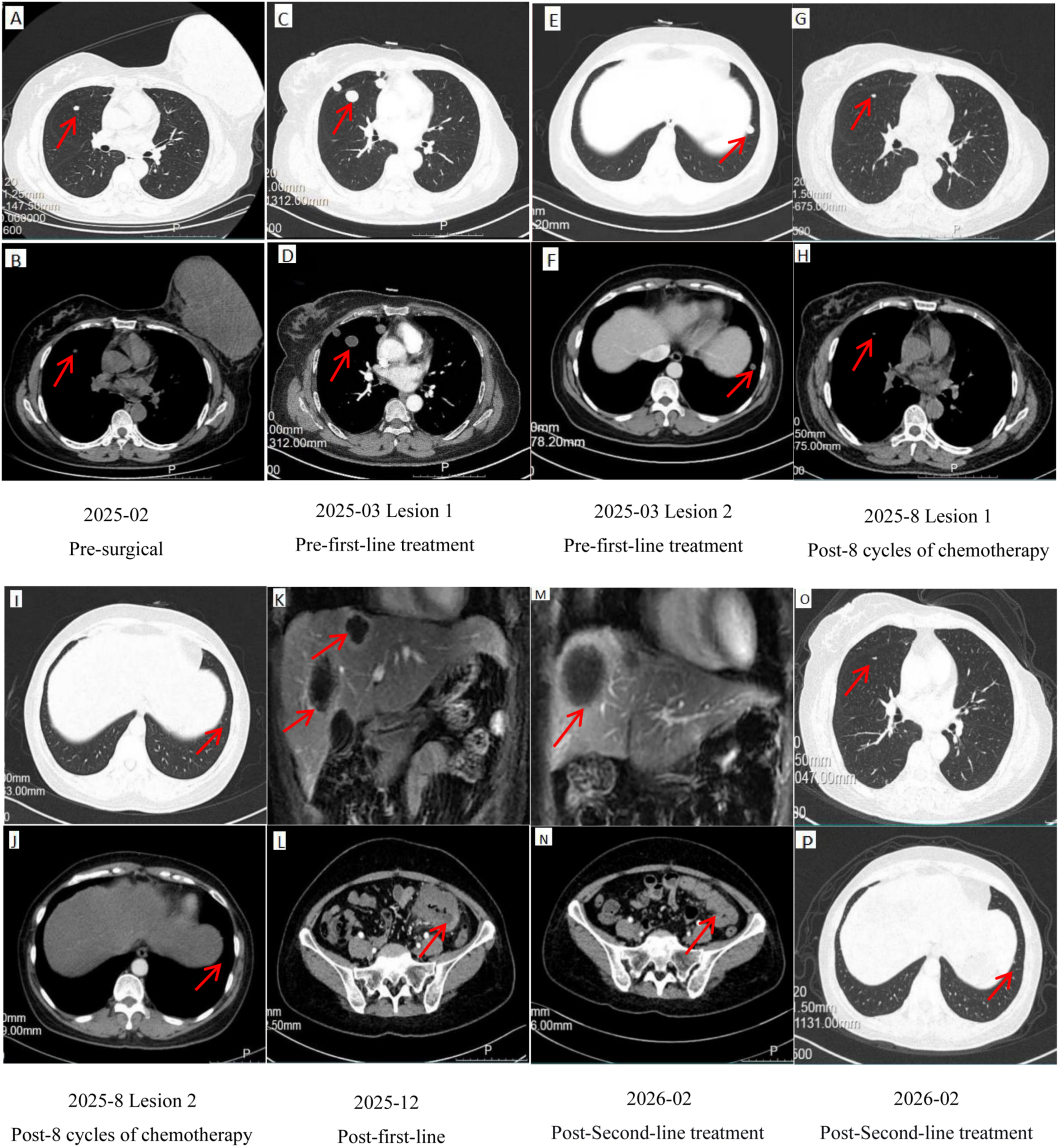
**(A, B)** CT images of the lung window **(A)** and the mediastinal window **(B)** on February 7, 2025; **(C, D)** CT images of the lung window **(C)** and the mediastinal window (D) for Lesion 1 pre-first-line treatment on March 18,2025; **(E, F)** CT images of the lung window **(E)** and the mediastinal window **(F)** for Lesion 2 pre-first-line treatment on March 18,2025. **(G, H)** CT images of the lung window **(G)** and the mediastinal window **(H)** for Lesion 1 post-8 cycles of chemotherapy on August 13,2025. **(I, J)** CT images of the lung window (I) and the mediastinal window **(J)** for Lesion 2 post-8 cycles of chemotherapy on August 13,2025. **(K, L)** MRI coronal images **(K)** of hepatic metastases after first-line progression on December 06, 2025 and CT images **(L)** of intestinal metastases on December 17, 2025. **(M, N)** Liquefactive necrosis of the liver on MRI **(M)** and reduction of intestinal metastatic lesions on CT **(N)** after two cycles of second-line therapy on February 3, 2026. **(O, P)** Lung lesions1 **(O)** and lung lesions 2 **(P)** after two cycles of second-line therapy on February 3, 2026.

On February 8, 2025, she underwent a left modified radical mastectomy plus axillary lymph node dissection. Intraoperative frozen section pathology indicated a malignant breast tumor. In the absence of preoperative histologic confirmation, surgical management followed standard oncologic principles for suspected malignant breast tumors, aiming to achieve complete tumor resection and accurate axillary staging to guide subsequent therapy. Histopathological examination (February 13, 2025) showed a single mass in the left breast, measuring approximately 16.0 × 15.0 × 9.5cm. The cut surface was gray–white to gray–red with extensive central necrosis; focal areas showed a fish-flesh-like appearance and soft consistency. Microscopically, low-power magnification revealed an infiltrative tumor border, marked stromal hypercellularity, and stromal overgrowth with obliteration of epithelial components in some regions. High-power examination demonstrated prominent stromal cytologic atypia and frequent mitotic figures (10–12 per 10 high-power fields, HPF). On the basis of hematoxylin-and-eosin morphology together with immunohistochemical findings, the diagnosis was malignant phyllodes tumor. Lymphovascular invasion was present, and perineural invasion was absent. No metastatic tumor was identified in the dissected left axillary lymph nodes (0 of 18). According to the AJCC 8th edition (2017), the pathological stage was pT4N0 (pM not assessed). Immunohistochemistry (**Figure 2**) demonstrated the following profile: ER (-), PR (-), CerbB-2 (-), Ki67 (+, ~80%), CK5/6 (-), SMA (-), desmin weakly (+), S100 (-), CD34 weakly (+), Rb (+), p53 (overexpression), SOX10 (-), BRAF V600E (-), CK8/18 (-), MyoD1 (-), GATA3 (-), p40 (-), BCL2 (+), CD117 (-), CD10 (+). Overall, these findings supported the diagnosis of malignant phyllodes tumor. Postoperative follow-up examinations, prompted by the treating physicians, on March 18, 2025 (**Figures 3C–F**), surveillance computed tomography suggested bilateral multiple pulmonary metastases, with the largest lesion measuring 2.0cm in maximum diameter. After implantation of a venous access port, systemic therapy was initiated with an ifosfamide-anthracycline regimen every 3 weeks: ifosfamide 2g on days 1 through 5 plus epirubicin 120 mg on day 1 for eight cycles. This regimen was selected based on the common use of anthracycline combined with ifosfamide in soft tissue sarcoma, and epirubicin was chosen instead of doxorubicin to reduce cumulative cardiotoxicity. Response assessment according to RECIST version 1.1 demonstrated a partial response (**Figures 3G–J**), with an approximately 90% reduction in tumor burden. After completion of chemotherapy, she began switch-maintenance therapy with oral anlotinib (12 mg once daily on days 1 through 14 of a 21-day cycle). On December 6, 2025, liver magnetic resonance imaging showed new metastatic lesions (**Figure 3K**) that were not present on the scan dated March 18, 2025. On December 17, 2025, contrast-enhanced computed tomography (**Figure 3L**) demonstrated a space-occupying lesion in the left lower abdomen involving the distal descending colon and small intestinal tract, which was considered metastatic. The patient experienced significant lower abdominal pain with an NRS score of 6 but without intestinal obstruction, perforation, or bleeding. The symptoms were well controlled after treatment with oral opioid analgesics. A multidisciplinary team (MDT) consultation was conducted for the colonic space-occupying lesion, and it was determined that there was no indication for emergency surgery or interventional therapy due to the absence of mechanical obstruction, perforation, or bleeding. Systemic antitumor therapy was considered the fundamental treatment to relieve the symptoms from the root cause. The patient underwent PD-L1 testing of the primary breast tissue, with a TPS of 1% and a CPS of 10. After MDT consultation, second-line treatment was selected as an individualized, off-label salvage regimen in metastatic MPT. During the MDT discussion, the SAGC and CBCSG-006 clinical studies were reviewed as indirect, non-MPT-specific references for related treatment components, rather than as direct evidence for this regimen in MPT (14, 15). The decision was based on the patient’s previous pulmonary disease control during anlotinib maintenance, rapidly progressive symptomatic abdominal disease, preserved performance status and organ function, and the feasibility of combining immune checkpoint blockade, antiangiogenic therapy, and gemcitabine–cisplatin chemotherapy. Accordingly, second-line combination therapy was initiated on December 24, 2025 in a 21-day cycle: anlotinib 12 mg on days 1 through 14, sintilimab 200 mg on day 1, gemcitabine 1.5g on days 1 and 8, plus cisplatin 110 mg on day 2. After two cycles, the treatment response was assessed as PR (marked reduction of the left lower abdominal lesion and liquefactive necrosis of the liver lesion) (**Figures 3M–P**). No recurrence has been detected in the chest wall to date (**Figure 1C**).

**Figure 4:**
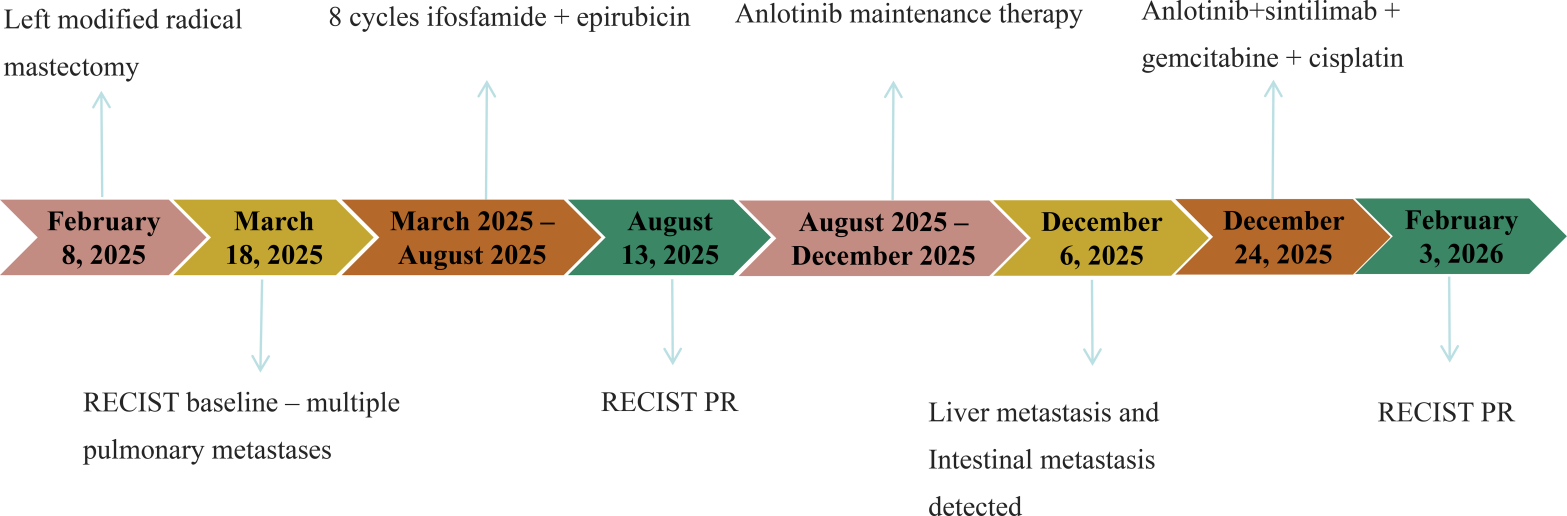
Patient treatment timeline. RECIST, Response Evaluation Criteria in Solid Tumors; PR, partial response.

At baseline, physical examination revealed no palpable lymphadenopathy, well-healed surgical scars, and no significant abnormalities on systemic examination. Prior to second-line therapy, the patient developed palpable hepatomegaly and tenderness in the lower abdomen without rebound tenderness. Throughout the follow-up period, her overall condition remained good. The differential diagnosis of this patient is shown in **Table 1**, and the treatment timeline is illustrated in **Figure 4**.

**Table 1:** Differential diagnosis of this patient.

Differential diagnosis	Clinical characteristics	Imaging features	Pathological features	Key distinguishing features in this case
Primary invasive breast carcinoma	Predominantly occurs in women aged 40–60 years; presents as hard, irregular mass; may have peau d’orange skin changes, nipple retraction or discharge; lymph node metastasis common	Mammography: irregular high-density mass with spiculated margins, possible microcalcifications; Ultrasound: hypoechoic, irregular shape, aspect ratio >1; MRI: rapid enhancement with washout	Malignant epithelial tumor; ER/PR may be positive; HER2 may be overexpressed; CK8/18, GATA3 positive; variable Ki-67 index	CK5/6,CK8/18, and GATA3 all negative, excluding epithelial origin; absence of lymph node metastasis (0/18) inconsistent with typical breast cancer metastatic pattern
Giant fibroadenoma	Predominantly occurs in adolescents and young women (<35 years); slow-growing, well-circumscribed, mobile mass; large tumors may cause skin expansion and venous distension without ulceration or fixation	Ultrasound: well-circumscribed hypoechoic mass with intact capsule, homogeneous internal echoes; Mammography: round or oval high-density mass with smooth margins; MRI: hyperintense on T2WI, homogeneous enhancement	Benign fibroepithelial tumor; epithelial and myoepithelial markers positive (CK, P63, SMA); CD34 may be positive; low Ki-67 index (usually <5%); no p53 mutation expression; BCL-2 often negative or weakly positive	Patient age 53 years inconsistent with typical presentation; 16-cm tumor with lymphovascular invasion; Ki-67 80%; malignant stromal overgrowth on pathology; absence of intact capsule—all inconsistent with benign features of fibroadenoma
Primary breast sarcoma	Occurs across all age groups; presents as rapidly enlarging giant mass; skin changes common (vascular dilation, violaceous discoloration); early hematogenous metastasis, lymph node metastasis uncommon	Ultrasound: giant hypoechoic mass with ill-defined margins, heterogeneous internal echoes; MRI: iso- or hypointense on T1WI, hyperintense on T2WI, heterogeneous enhancement; necrosis and hemorrhage may be present	Malignant tumor of mesenchymal origin, lacking epithelial markers (CK, GATA3 negative); expresses corresponding mesenchymal differentiation markers: angiosarcoma (CD31, CD34, ERG positive), liposarcoma (S-100 may be positive), leiomyosarcoma (SMA, desmin positive)	Definite biphasic architecture with benign epithelial and malignant stromal components in this case; simple sarcoma lacks this biphasic pattern; desmin only weakly positive and SMA negative, not supporting leiomyosarcoma; no adipose differentiation, excluding liposarcoma; no vascular differentiation features, excluding angiosarcoma

ER, estrogen receptor; PR, progesterone receptor; HER2, human epidermal growth factor receptor 2; CK, cytokeratin; GATA3, GATA binding protein 3; SMA, smooth muscle actin; CD, cluster of differentiation; Bcl-2, B-cell lymphoma 2.

Written informed consent was obtained from the patient for publication of her clinical, imaging, and pathological data. The study was approved by the Ethics Committee of Jiangsu Province (Suqian) Hospital and conducted in accordance with the Declaration of Helsinki.

## Discussion

Malignant phyllodes tumors (MPTs) of the breast are rare fibroepithelial neoplasms with a propensity for early hematogenous dissemination. This patient exhibited early explosive bilateral pulmonary metastasis following curative resection, with large tumor size (pT4) and lymphovascular invasion representing well-recognized high-risk features for hematogenous spread (4, 5, 16, 17). A small preoperative pulmonary nodule likely reflected pre-existing micrometastatic disease, and the failure to identify occult metastasis before surgery delayed the initiation of systemic therapy. Against this clinical background, systemic treatment decision-making for metastatic MPT largely follows established recommendations for soft-tissue sarcoma.

According to the NCCN guidelines for breast cancer and soft-tissue sarcoma, the combination of ifosfamide and anthracyclines (epirubicin) represents a standard first-line regimen for soft-tissue sarcoma with category 2A evidence (6–8, 18). In this case, the patient was relatively young, had no major comorbidities, and maintained an excellent performance status, which supported excellent chemotherapy tolerability and allowed administration of intensive chemotherapy. Given the early postoperative bilateral pulmonary metastases, the regimen of ifosfamide plus epirubicin was administered and showed marked activity; after eight cycles, the lung lesions met criteria for a partial response (>90% reduction). Given that the patient did not achieve a clinical complete response (cCR) on radiological evaluation and due to the maximum cumulative dose constraint of anthracyclines, the treatment regimen was transitioned to oral anlotinib as maintenance therapy. Anlotinib is a multitarget receptor tyrosine kinase inhibitor with activity against several angiogenesis- and growth-related pathways, including VEGFR, PDGFR, FGFR, and c-KIT. The selection of anlotinib for maintenance was based primarily on efficacy data in advanced soft-tissue tumors. In recent reports (9, 19, 20), anlotinib maintenance therapy following first-line chemotherapy has demonstrated favorable but preliminary disease control activity in advanced soft-tissue sarcomas, with no specific data supporting its application in malignant phyllodes tumors. In this metastatic MPT patient, the pulmonary lesions remained stable during anlotinib maintenance, but new hepatic and colorectal metastases emerged at 4 months of treatment, meeting RECIST criteria for progressive disease.

Newly progressive lesions should be considered for repeat biopsy to define molecular evolution and guide subsequent treatment. A strategy combining local approaches (e.g., transarterial embolization, ablation, or stereotactic radiotherapy for liver lesions) with adjustment of systemic therapy (e.g., alternative chemotherapy, pathway-directed targeted agents, immune checkpoint inhibitors, or clinical-trial participation) may be reasonable. The patient declined a liver biopsy. Due to the presence of multiple metastatic lesions, radiotherapy offered limited benefit. Immune checkpoint inhibitors have demonstrated clinical activity in several sarcoma subtypes (12, 13), yet evidence in MPTs remains limited. In the present case, immunohistochemical analysis revealed PD-L1 expression with a combined positive score (CPS) of 10, providing a biological rationale for immunotherapy. Nevertheless, single-agent immune checkpoint inhibitor therapy yields limited efficacy in patients with moderate CPS expression. Following thorough assessment of KPS performance status and safety profile, a second-line regimen comprising sintilimab, anlotinib, gemcitabine, and cisplatin was initiated. Sintilimab is a highly selective humanized monoclonal antibody targeting the PD-1 receptor on T cells, thereby blocking its interaction with PD-L1 and reversing tumor-induced immunosuppression. This reverses the immunosuppressive microenvironment, promotes T−lymphocyte activation, increases the CD4+/CD8+ and Th1/Th2 ratios, reduces Treg levels, and restores tumor immune surveillance, thereby preventing tumor immune escape and exerting antitumor effects (21–23). As an anti−angiogenic agent, anlotinib normalizes tumor vasculature, enhances immune cell infiltration, and modulates immune cell composition within tumor tissues to alleviate immunosuppression in the tumor microenvironment (24). Preclinical studies have demonstrated that the combination of sintilimab and anlotinib reduces the activity of myeloid−derived suppressor cells and regulatory T cells, reshapes the tumor microenvironment, converts an immunosuppressive state into an immune−permissive phenotype, normalizes tumor blood vessels, promotes T−cell infiltration into tumors, strengthens antitumor immune function, and blocks immunosuppressive signals through multiple pathways to augment antitumor activity (25). Accumulating evidence indicates that combined therapy with chemotherapy and immune checkpoint inhibitors (ICIs) enhances the ability of the host immune system to recognize and eliminate tumor cells while reducing immunosuppression induced by the tumor microenvironment to achieve synergistic clinical efficacy (26). Following two cycles of treatment, the patient achieved a partial response (PR), with marked shrinkage of the abdominal lesions and liquefactive necrosis in the hepatic lesions. Clinically, the patient’s abdominal pain was completely resolved, and opioid analgesics were discontinued, which significantly improved the patient’s quality of life. Reports describing the use of immune checkpoint inhibitors in malignant phyllodes tumors are exceedingly limited (27); therefore, the marked response observed in this patient represents a noteworthy clinical observation.

It should be noted that this study has several limitations. First, PD-L1 expression was only detected in the primary tumor specimen without re-biopsy of metastatic lesions, which may compromise the predictive accuracy of ICI treatment response. Second, comprehensive molecular profiling (e.g., NTRK, RET) was not performed prior to first-line chemotherapy. Third, it remains unclear whether PD-L1 CPS-positive patients could achieve superior outcomes if ICI therapy is administered as first-line treatment. Fourth, although anlotinib was continued in the second-line ICI plus anti-angiogenic combination regimen, high-level evidence supporting the continuation of tyrosine kinase inhibitors after RECIST-defined disease progression remains limited in sarcoma patients. Finally, the gemcitabine plus cisplatin chemotherapy backbone was selected based on individualized clinical judgment rather than formal guideline recommendations, and high-level evidence specifically for malignant phyllodes tumors (MPTs) is still lacking to validate this regimen.

## Conclusion

This case highlights the therapeutic complexity and biological heterogeneity of metastatic malignant phyllodes tumors of the breast. Although anthracycline-based first-line chemotherapy produced marked tumor regression, disease control was not sustained during subsequent antiangiogenic maintenance therapy. In this PD-L1–positive tumor (CPS 10), immune checkpoint inhibitor–based combination therapy was followed by shrinkage of the abdominal lesion, liquefactive necrosis of hepatic lesions, and complete relief of abdominal pain, supporting further investigation of immunotherapy in selected patients with metastatic MPT. Multicenter studies incorporating molecular profiling are needed to define clinically relevant subgroups and to guide individualized treatment strategies for this rare disease.

## Data Availability

The raw data supporting the conclusions of this article will be made available by the authors, without undue reservation.
